# Open energy system modelling to support the European Green Deal

**DOI:** 10.12688/f1000research.121619.1

**Published:** 2022-05-16

**Authors:** Diana Süsser, Bryn Pickering, Ludwig Hülk, Stefan Pfenninger

**Affiliations:** 1 Institute for Advanced Sustainability Studies (IASS), Potsdam, Germany; 2 Institute for Environmental Decisions (IED), ETH Zurich, Zurich, Switzerland; 3 Reiner Lemoine Institut (RLI), Berlin, Germany; 4 Faculty of Technology, Policy and Management, TU Delft, Delft, The Netherlands

**Keywords:** Energy modelling, energy models, open source, transparency, EMP-E

## Abstract

Energy models are used to explore decarbonisation pathways and potential future energy systems. In this editorial, we comment on the importance of energy system modelling and open tools to inform policymaking in the context of the European Green Deal. We also summarise the seven contributions to the special collection on Energy Systems Modelling, among which are papers that have been presented at the Energy Modelling Platform for Europe (EMP-E) 2021 conference. The presented research advances current modelling approaches and supports energy modelling with open tools and datasets.

To fulfil its obligations under the Paris Agreement, the European Union is committed to reducing its greenhouse gas emissions by 55% from 1990 levels by 2030 and achieving climate neutrality by 2050 ( European Commission, 2020). Climate neutrality implies complete decarbonisation of energy, and therefore requires a profound transformation. The *European Green Deal* lays the foundation for this and can be understood as a compass for transforming the EU economy into a resource-efficient, competitive and climate neutral economy ( European Commission, 2019).

Energy system models and tools can be used to explore decarbonisation pathways and test the outcomes of interconnected choices about the design of our future energy systems. They can support decision-makers in assessing the benefits, costs, risks, and trade-offs of strategies and investments related to the transition to a climate-neutral society. To make models more relevant for policymaking, they need to be (i) better tailored to the needs of those who will use them, and (ii) “opened up” to become eventually more understandable ( Cao *et al.*, 2016; Pfenninger, 2017; Pfenninger *et al.*, 2018).

First, decision-makers seek models that go beyond the techno-economic sphere and take into account all relevant aspects of the energy transition, including social, political, and environmental aspects ( Süsser *et al.*, 2022). Yet, factors such as social preferences and policy dynamics are entirely ignored most of the time, or only considered as an exogenous narrative ( Krumm, Süsser and Blechinger, 2022). Therefore, existing approaches need to be extended and new modelling approaches need to be developed so that the models can better represent real-world conditions and developments. This special collection on Energy Systems Modelling includes new modelling methods and datasets that address energy sufficiency policies ( Best *et al.*, 2022) and peer-to-peer trading ( Perger and Auer, 2022) in modelling, as well as assess energy infrastructure needs ( Arduin *et al.,* 2022).

Second, much of the data and results from energy models need to be made as open and accessible as possible to be useful to different users ( Bazilian *et al.*, 2012; Süsser *et al.*, 2022). Transparency is key to ensuring that the implications and limitations of each model are fully understood such that they can be better applied in the context of potential policy options ( Hülk *et al.*, 2018). Previous research has shown that open source and transparent energy model developments allow modellers and users to discuss different needs and create a shared understanding of modelling ( Pfenninger *et al.*, 2018; Ben Amer *et al.*, 2020). Furthermore, open exchange about different modelling aspects creates confidence and trust in models ( Süsser et al., 2022). These are reasons why there is a paradigm shift towards open methods in energy modelling projects ( Morrison, 2018), as also reflected in the contributions of this special collection. It presents new open datasets ( Sterl *et al.*, 2022) and new methods for citizen-driven data collection ( del Cañizo *et al.*, 2021) as well as a better processing of open data sets ( Fleischer, 2022) to increase the use of open data in modelling. Additionally, the collection presents a new tool for the analysis and visualisation of results ( Huppmann *et al.*, 2022) to streamline the postprocessing pipeline.

The special collection on Energy Systems Modelling, running concurrently on F1000Research and Open Research Europe, presents various contributions that advance current modelling approaches and support energy modelling with open tools. This collection includes papers from the Energy Modelling Platform for Europe (EMP-E) 2021 conference, which were discussed with researchers and decision-makers. The annual EMP-E conference brings together the European energy modelling community over a three-day period in a forum for an intensive and diverse exchange of research and modelling practice ( Müller, Gardumi and Hülk, 2018). The fifth edition of this annual conference discussed the role of models for setting and achieving policy targets, the linking of different sectors in energy models and specific modelling challenges, including the representation of social aspects in models and model transparency. In addition, the conference offered scientists and decision-makers the opportunity to learn about and test various modelling tools in dedicated “skill workshops”.

The Energy Systems Modelling collection currently includes seven diverse scientific contributions, ranging from Software Tool Articles and Data Notes to Research Articles. Here, we provide a summary of the individual contributions that have been published in the collection to date.

Modelling country or continent-wide multi-energy systems at a high resolution is a data intensive task. In Europe, pre-processed data is relatively abundant online. However, coordinating and combining this data is not straightforward. Fleischer (2022) presents EU-SES, a software package to process the myriad online datasets available to describe energy demand and supply for up to 28 European countries. With EU-SES a user can first build a standardised dataset for their geographic area of interest, containing 19 data variables with a possible hourly temporal resolution and sub-national spatial resolution. These variables describe the location of power plants, the physical limits on renewable technology capacity and hourly productivity, and the hourly demand for heat and electricity. With this dataset constructed, a user can then build their preferred representation of the system by aggregating to regions. These regions could match administrative units or be derived by EU-SES based on clustering areas to match areas with similar population and renewable resource potential. This clustering algorithm is a particularly unique methodological advancement in the processing of European datasets, with the potential to maintain the spatio-temporal features of the underlying data without compromising on overall model size.

The work of an energy systems analyst is not over when they have completed their model runs. The processing and visualisation of results remains a time-consuming hurdle. With *pyam*, Huppmann *et al.* (2022) hope to solve this issue by bridging the gap between different data formats and modelling frameworks to provide a way for all energy system and integrated assessment modellers to easily process and visualise their data. *pyam* is a Python toolbox with clear design principles, based on lessons learned from several Horizon 2020 projects and the Integrated Assessment Modeling Consortium (IAMC) data format. With strong links to the well-known *pandas* package, both seasoned and novice Python users can quickly get to grips with pyam’s functionality. It is already proving valuable as part of the IPCC’s Sixth Assessment Report (AR6) and the Horizon 2020 project *European Climate and Energy Modelling Forum* (ECEMF).

To model renewable energy in energy systems, modellers require data on renewable resource profiles with high spatiotemporal resolution. This data is available for solar power and wind power but was not available for hydropower across continents. Therefore, Sterl *et al.* (2022) developed a new open-access database, the “African Hydropower Atlas”, which contains seasonal hydropower generation profiles for nearly all existing and several hundred future hydropower plants in Africa. The atlas builds on continental-scale hydrological modelling in combination with detailed technical databases of hydropower plant characteristics. The atlas provides an important contribution to facilitate modelling of hydropower generation across Africa.

Citizen science has gained increasing relevance in bringing science and society closer together. del Cañizo *et al.* (2021) present a case study of the citizen science initiative “Generation Solar”. The aim of the initiative is to co-create an open database of PV installations, including their technical characteristics, and an online map to visualise the locations. The research team involved citizens, companies and public institutions in the research process from the beginning to co-develop research questions and identify interests and concerns. If Generation Solar is successful, it will provide valuable, openly licensed data for a wide range of researchers, including energy modellers.

Energy sufficiency aims to reduce electricity use, substitute technical services or adjust service use, thereby reducing the overall energy consumption of a households or country. It is rarely considered in energy modelling and is also underrepresented in energy and climate policy, unlike energy efficiency and renewable energy ( Zell-Ziegler *et al.*, 2021). Best *et al.* (2022) conducted an extensive literature review of European and national sufficiency policies to fill a gap in existing databases. The “Energy Sufficiency Policy Database” presents almost 300 policy instruments from seven sectors. They show that one third of the sufficiency measures have been introduced in the transport sector. The sufficiency policies go beyond the ban of products or information tools and address issues of ‘avoid’, such as reducing trips, and ‘shift’, such as shifting from high-energy to lower-energy modes. In the energy sector, measures concentrate on the overall policy goal to ‘reduce energy consumption’ with instruments like ‘subsidise energy savings’. This database provides insights for energy modelling and sufficiency policy options for the decarbonisation of the energy system.

Electricity generation is increasingly decentralised and therefore provides communities the opportunity to participate in the generation, storage, and trading of electricity. The research article by Perger and Auer (2022) deals with the participation of energy communities in local electricity markets via peer-to-peer trading. They apply a bi-level optimisation model to investigate how the preferences of existing community members influences the kind of new members which are allowed to join, and the impact of those new members on the system performance. First, the current welfare (emissions, system cost, etc.) of the existing community is captured. Next, the bi-level optimisation defines the kind of new member which would maximise the community’s welfare in the future using the existing community’s welfare as a benchmark. Two primary types of communities are defined, with different objectives to achieve welfare maximisation: environment-oriented (CO _2_ emissions minimisation) and profit-oriented (financial cost minimisation). They show that these two communities prefer very different kinds of new members. Profit-oriented communities would prefer new prosumers with high electricity demands and little existing PV capacity, since they will more likely increase the overall profits of the community. Conversely, environment-oriented communities prefer their new members to have low demands and high PV capacity. The methods described in this paper could be used by energy communities to ensure that their selection processes guarantee long-term community performance.

For a climate neutral future by 2050, the cross-border energy infrastructure needs to be assessed and quantified. Arduin *et al.* (2022) used a European energy system model to perform a detailed scenario analysis focussing on the electricity, methane and hydrogen transport. The multi-energy model Artelys Crystal Super Grid optimises on national granularity and in an hourly time resolution for the year 2050. The study concludes that large investments in electricity infrastructure are needed, especially between Central European countries. In addition, existing methane pipelines should be repurposed and supplemented by new hydrogen pipelines. Arduin *et al.* (2022) also find that hydrogen infrastructure requirements are highly dependent on the geographical allocation of renewables in Europe. The key policy recommendation from this scenario analysis is that detailed cost-benefit analyses, especially for power-to-x projects, should examine the impacts of a consistent deployment of renewable energy and electrolysers to avoid unnecessary investments in wires and pipelines. There is no need for additional investments in methane infrastructure (besides repurposing for hydrogen) in any scenario to meet the targets to support 1.5°C scenarios in the European Union.

The research presented in this collection makes valuable contributions to advance the modelling of energy systems. We strongly believe that an open modelling community will enable exchange among modelling teams and between the community and decision-makers. Such exchanges will foster collaboration and trust between modellers and users and thus increase the relevance of models in guiding Europe’s transition to climate neutrality.

We, the collection advisors, would like to thank the European Commission for supporting the Energy Modelling Platform for Europe and for funding the participating research projects AURES II, MAGNITUDE, NAVIGATE, OpenENTRANCE, Plan4RES, PLANET, SPINE, WHY and, especially the coordinating project SENTINEL. We would also like to thank the reviewers for taking the time to review the contributions. Finally, we would like to thank F1000Research and Open Research Europe for offering this collection and assisting us with the submission process and outreach. The Energy Systems Modelling collection remains open for further contributions to F1000Research and Open Research Europe.

## Data availability

No data are associated with this article.
